# Supplementary material for: Molecular basis for disassembly of an importin:ribosomal protein complex by the escortin Tsr2
Source: Nat Commun. 2018 Sep 10;9:3669. doi: 10.1038/s41467-018-06160-x (PMC6131548; doi:10.1038/s41467-018-06160-x)
Supplement: Supplementary file 1 — Supplementary Information [file 41467_2018_6160_MOESM1_ESM.pdf]

Molecular basis for disassembly of an importin:ribosomal protein complex by the escortin Tsr2

Schütz et al.

Supplementary Data

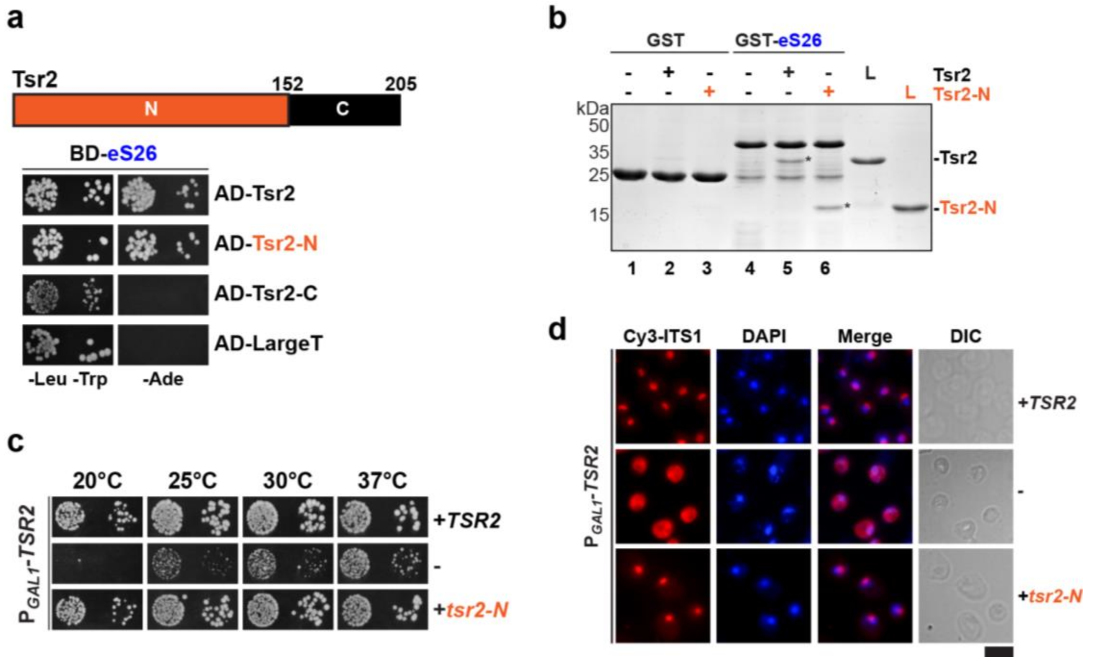

Supplementary Figure 1

The N-terminal domain of Tsr2 is sufficient for its function *in vivo*. (**a**) Tsr2 and Tsr2$^{1\text{-}152}$ (Tsr2-N) but not Tsr2$^{153\text{-}205}$ (Tsr2-C) interacts with eS26 in a yeast two-hybrid assay. Upper panel: Scheme showing domain architecture of Tsr2. Tsr2$^{1\text{-}152}$ (Tsr2-N) in orange, Tsr2$^{153\text{-}205}$ (Tsr2-C) in black. Lower panel: Plasmids encoding the indicated *GAL4* DNA-binding domain *(BD)* and *GAL4* activation domain *(AD)* fusion proteins were transformed into the yeast reporter strain NMY32. Transformants were spotted in 10-fold serial dilutions onto SD-Leu-Trp (-Leu-Trp) or SD-Ade (-Ade) and incubated at 30°C for 4 days. Growth on SD-Ade indicates a strong two-hybrid interaction. The SV40 Large T antigen served as negative control for these analyses. (**b**) Tsr2 and Tsr2-N directly bind eS26 *in vitro*. L= input (1:10 diluted). (**c**) Tsr2-N is sufficient to support yeast growth. The conditional P$_{GAL1}$-*TSR2* strain was transformed with empty vector or vector with *TSR2 WT* or *tsr-N*. Transformants were spotted in 10-fold dilutions on repressive glucose containing media and grown at indicated temperatures for 2-4 days. (**d**) Tsr2-N is sufficient for 20S pre-rRNA processing. P$_{GAL1}$-*TSR2* cells transformed with WT *TSR2* or *tsr2-N* were grown at 30°C in glucose containing media to mid-log phase. Localization of 20S pre-rRNA was analyzed by FISH using a Cy3-labeled oligonucleotide complementary to the 5′ portion of ITS1 (red). Nuclear and mitochondrial DNA was stained with DAPI (blue). Scale bar = 5 µm.

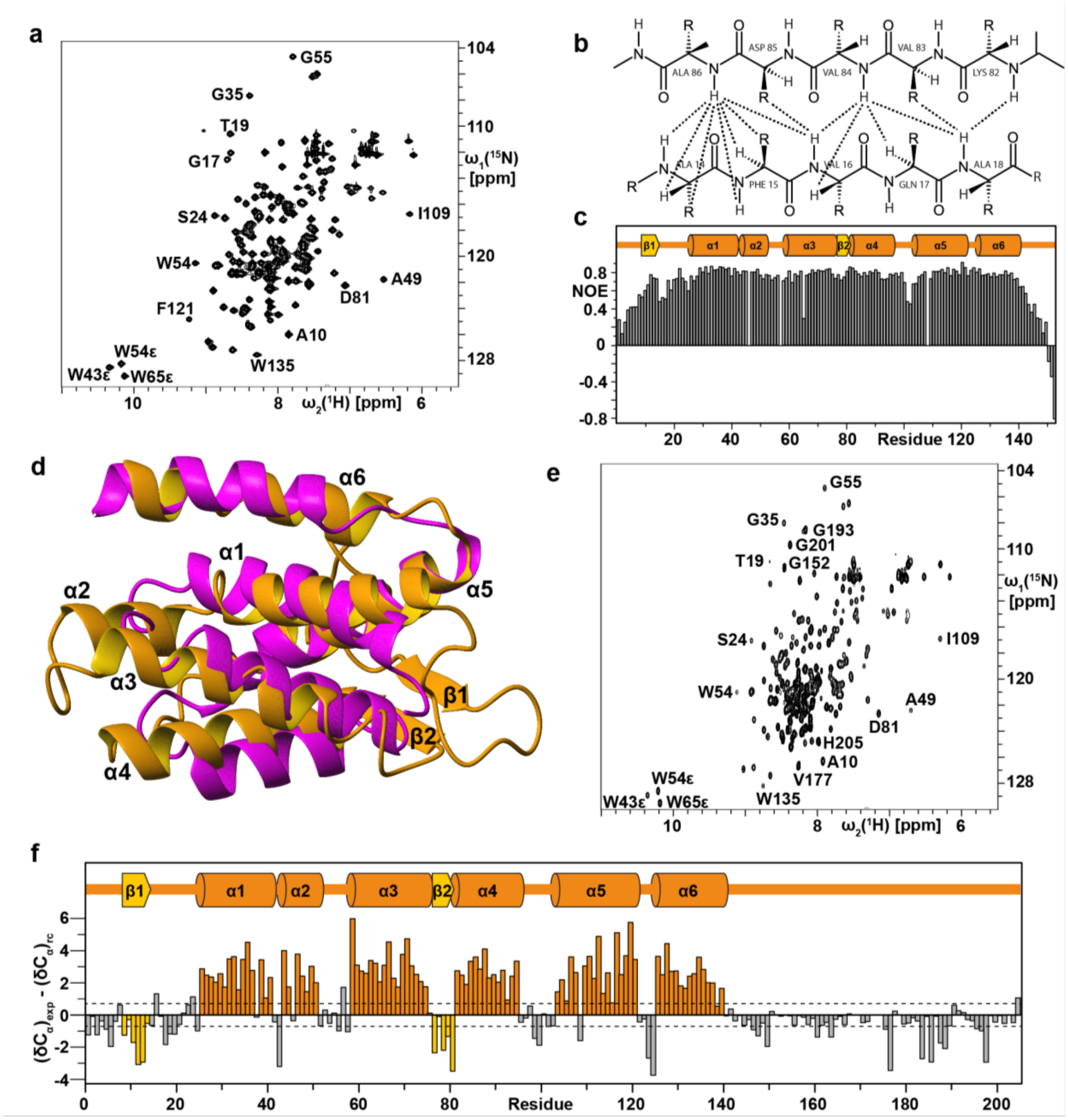

Supplementary Figure 2

NMR analysis of Tsr2-N and full-length Tsr2. (**a**) 2D [^{1}H,^{15}N]-HSQC spectrum of Tsr2-N recorded at 293.15 K. The assignments of a number of characteristic resonances are indicated. (**b**) Manual assignment of interstrand ^{1}H,^{1}H-NOEs (dotted lines) to confirm the presence of β-sheets in the solution structure Tsr2-N. (**c**) ^{15}N{^{1}H} NOE experiment of Tsr2-N recorded at 293.15 K and 700 MHz. (**d**) Superposition of cartoon representations of Tsr2-N (orange, lowest energy conformer) and Nab2 (magenta, PDB-2JPS) based on minimization of the rmsd between residues 26-43, 61-74, 82-92, 101-121, 129-142 of Tsr2-N and residues 4-21, 27-40, 46-56, 60-80, and 85-98 of Nab2. (**e**) 2D [^{1}H,^{15}N]-HSQC spectrum of full-length Tsr2 recorded at 303.15 K. Assignments of various characteristic signals are shown. (**f**) Secondary structure analysis of full-length Tsr2 based on the deviation of the assigned ^{13}Cα shifts from their respective random coil values[1]. The derived α-helices and β-strands are indicated with orange and yellow bars, respectively, and the dotted lines indicate the value of the standard deviation values of 0.7 ppm and -0.7 ppm.

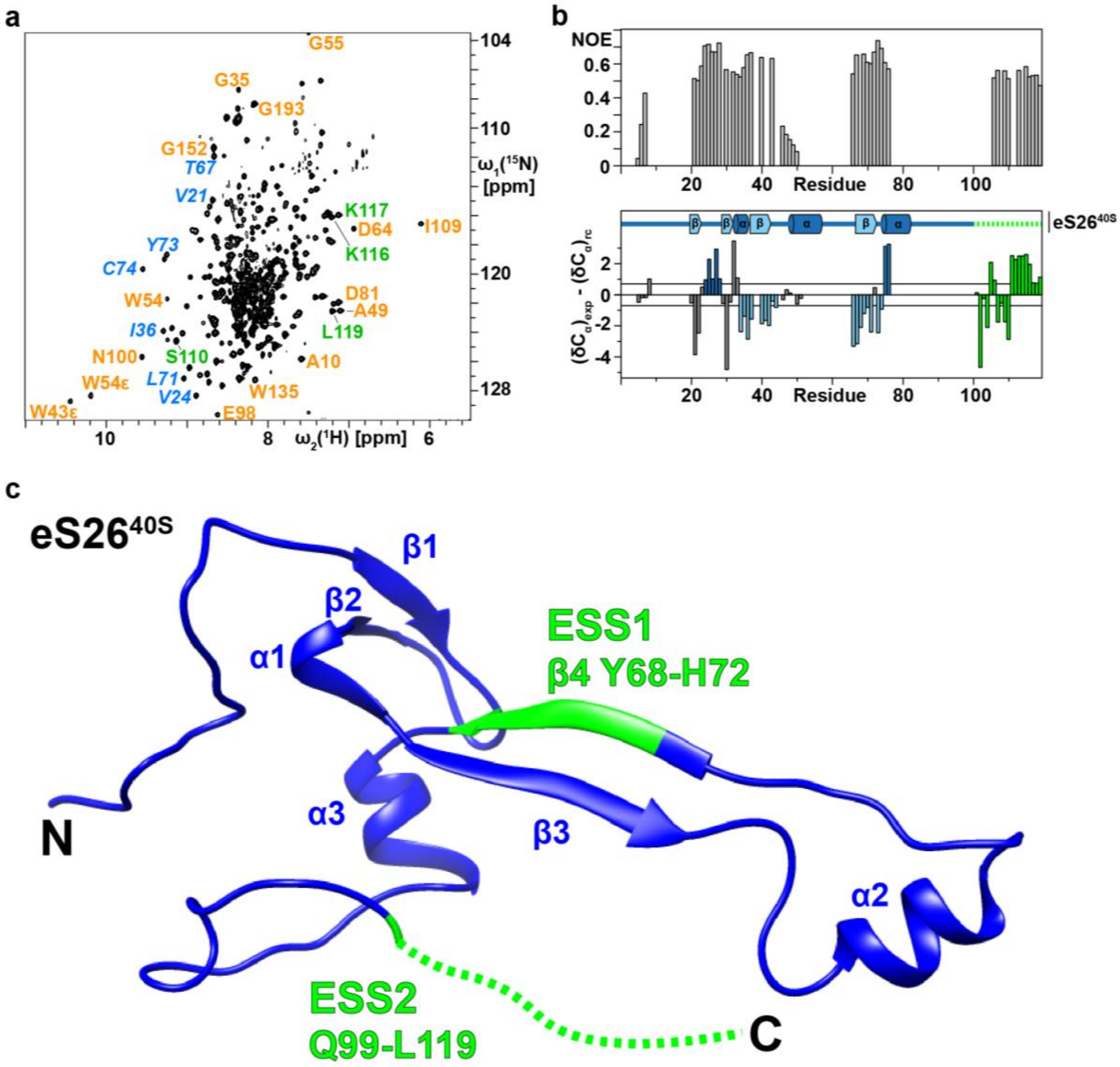

Supplementary Figure 3

NMR analysis of Tsr2 in complex with eS26. (**a**) 2D [^{1}H,^{15}N]-TROSY spectrum of full-length Tsr2 in complex with eS26 recorded at 303.15 K. The assignments of characteristic resonances of Tsr2 and eS26 are indicated with orange (Tsr2) and green (eS26) letters, respectively. (**b**) NMR analysis of full-length eS26 in complex with Tsr2. The ^{15}N{^{1}H} NOE data of eS26 in complex with Tsr2 shows that the conformational mobility for most of the assigned residues is similar to a folded protein, and the ^{13}Cα chemical shift deviations from random coil values[1] show that these regions have secondary structure similar to that observed when eS26 is part of the ribosome. Schematics of secondary structure observed for eS26 in the 40S particle of the ribosome and determined from ^{13}Cα shifts when it is bound to Tsr2 are shown at the top. The C-terminal residues 99–119 of eS26 (ESS2), which are not visible in the X-ray structure of the eukaryotic ribosome possess low conformational mobility and Cα shifts indicate the formation an α-helix comprising residues 111–119. **(c)** X-ray structure of yeast eS26 as bound in the mature 40S ribosome (PDB-4V88)[2].

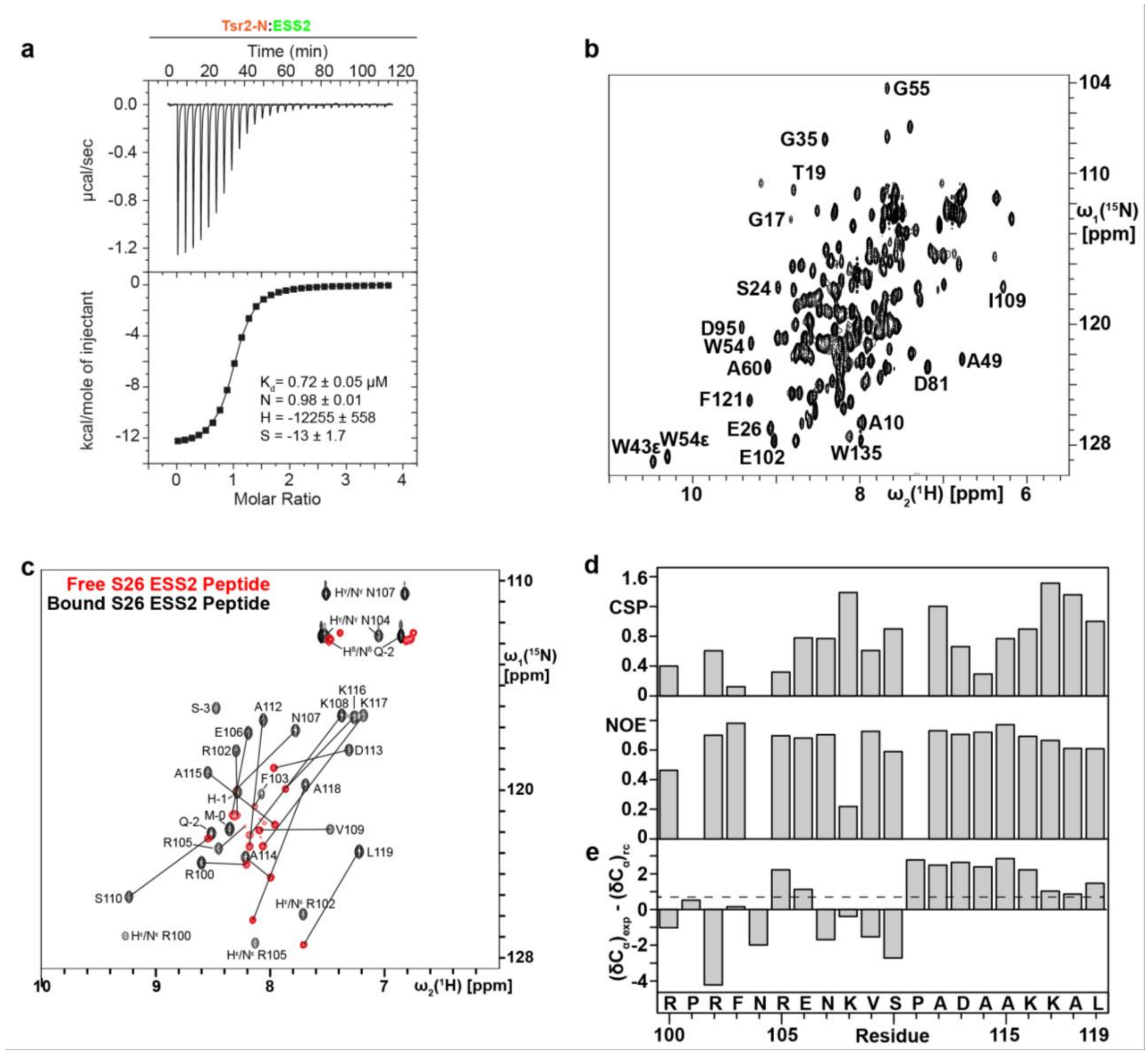

Supplementary Figure 4

Analysis of the interaction of Tsr2-N and the ESS2 peptide. (**a**) An ITC measurement with yeast Tsr2-N and ESS2 indicates a K_d of 0.7 µM. (**b**) 2D [^{1}H,^{15}N]-HSQC spectrum of ^{15}N-labeled Tsr2-N in complex with unlabeled ESS2 recorded at 293.15 K. The assignments of a number of characteristic Tsr2-N resonances are indicated. (**c**) Superposition of 2D [^{1}H,^{15}N]-HSQC spectra of free ^{15}N-labeled ESS2 (red signals) and in complex with unlabeled Tsr2-N (black signals) at 293.15 K. The resonance assignments of the bound conformation are indicated and connected by a line to the corresponding signals in the free form. Labels with superscripts denote sidechain resonances. The resonances for the expression tag are denoted according to their sequential position with respect to residue R100; S-3, Q-2, H-1 and M0 (**d**) Combined ^{1}H and ^{15}N chemical shift perturbation (CSP) and {^{1}H}^{15}N NOE values of the ESS2 amide resonances upon binding to Tsr2-N are indicated in upper and lower panels respectively. (**e**) Secondary structure analysis of individual ESS2 residues in the complex with Tsr2-N based on the ^{13}Cα shift deviation from random coil values. The dotted line indicates a standard deviation value of 0.7 ppm.

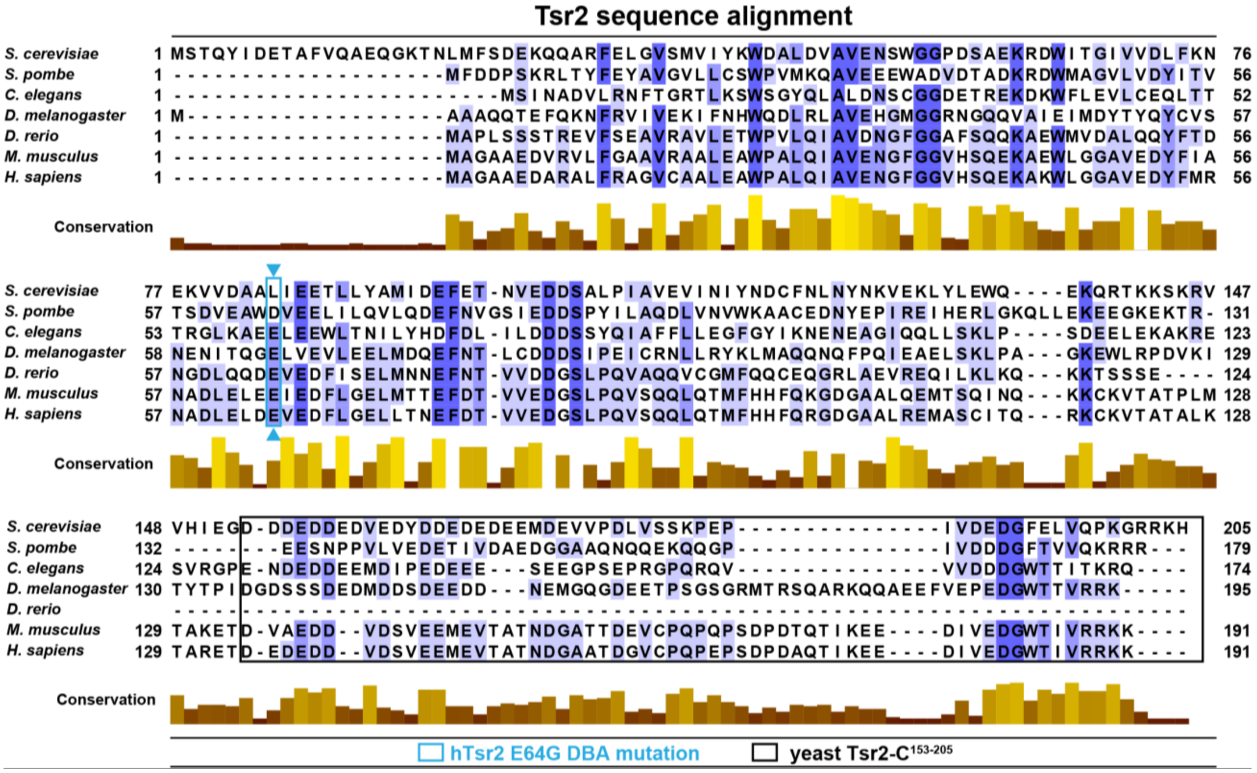

Supplementary Figure 5

Sequence conservation of Tsr2 in eukaryotes. Alignment was performed for indicated eukaryotic organisms[3].

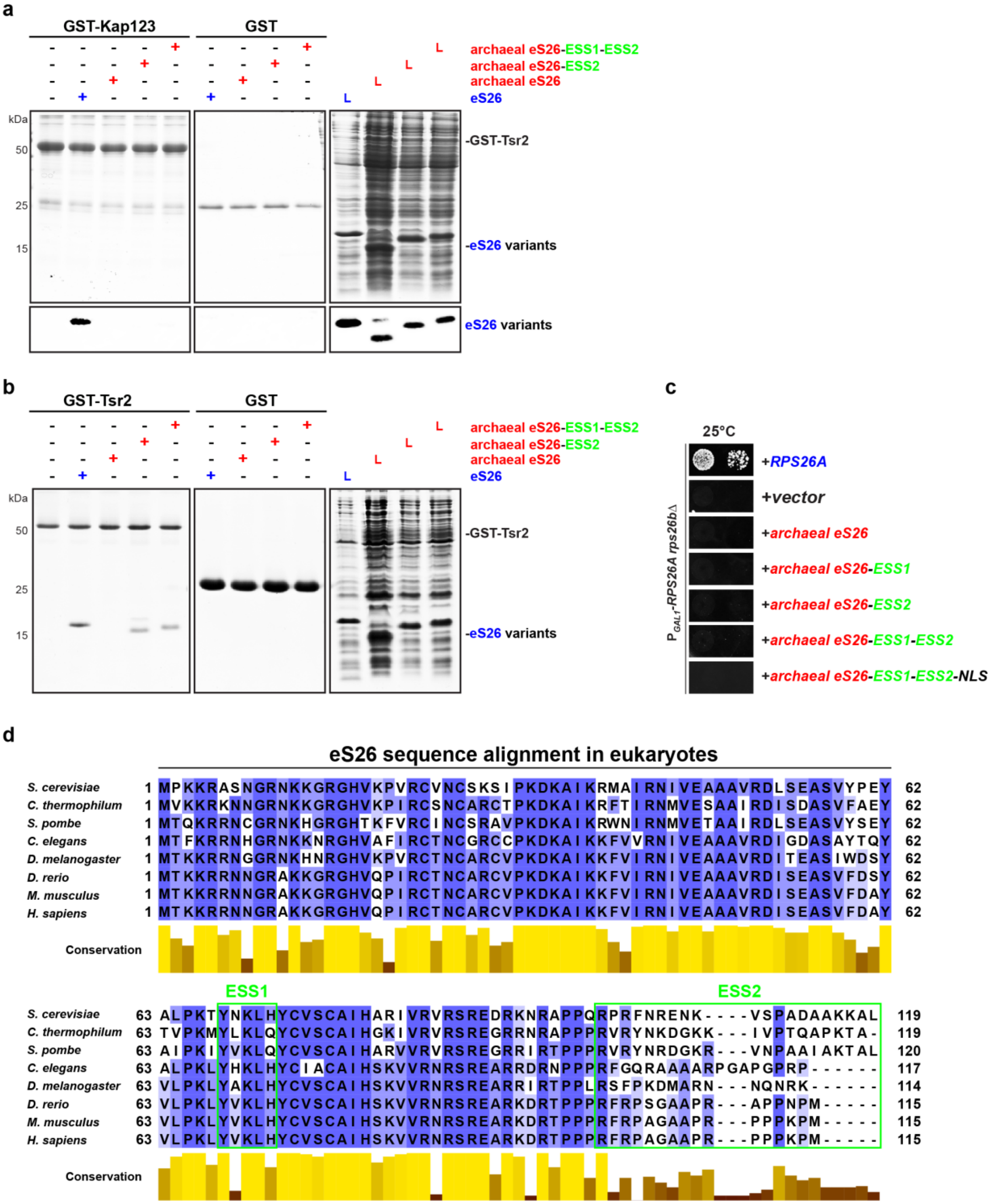

Supplementary Figure 6

Archaeal eS26 does not interact with importin Kap123 and the escortin Tsr2 (**a**) GST-Kap123 was immobilized on Glutathione Sepharose and incubated with FLAG-tagged yeast eS26 or FLAG-tagged archaeal eS26 from *Sulfolobus solfataricus* containing ESS1 and ESS2 insertions as indicated in Fig. 1c. Bound proteins were analyzed by Coomassie Blue staining and Western analyses used the α-FLAG antibody. L= input (1:10 diluted). **(b)** GST-Tsr2 was immobilized and incubated with eS26 variants as above (**c**) Archaeal eS26 variants that bind Tsr2 *in vitro* are unable to complement the lethality of the eS26-depletion strain. The conditional P_{GAL1}-*RPS26Arps26bΔ* strain was

transformed with the indicated variants of archaeal eS26 and spotted in 10-fold dilutions on repressive glucose-containing media and grown at 25°C for 4 days. **(d)** Sequence alignment of eS26 in eukaryotic organisms[3].

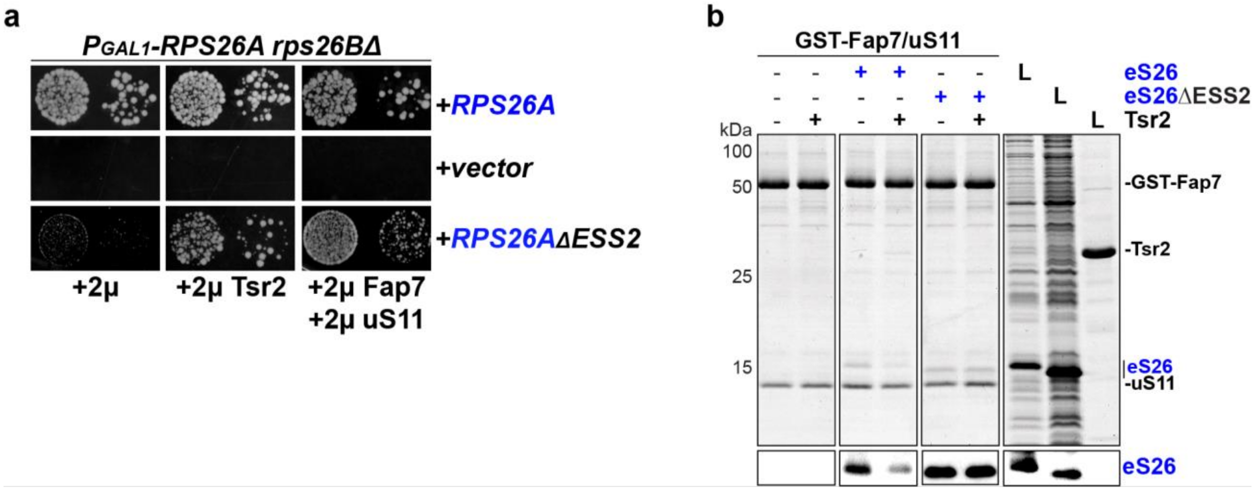

Supplementary Figure 7

Fap7:uS11 bypasses the need for Tsr2 in an ESS2-independent manner. **(a)** Overexpression of Tsr2 or Fap7:uS11 rescues growth impairment of eS26ΔESS2. The conditional P$_{GAL1}$-*RPS26Arps26b*Δ strain was transformed with the indicated 2µ high-copy plasmids and spotted in 10-fold dilutions on repressive glucose-containing media and grown at 25°C for 4 days. **(b)** eS26ΔESS2 is efficiently recruited to Fap7:us11. GST-Fap7:uS11:eS26 or GS-Fap7:uS11:eS26ΔESS2 was immobilized on Glutathione Sepharose and incubated with Tsr2. Bound proteins were analyzed by Coomassie Blue staining and Western analyses against FLAG-tagged eS26. L= input (1:10 diluted).

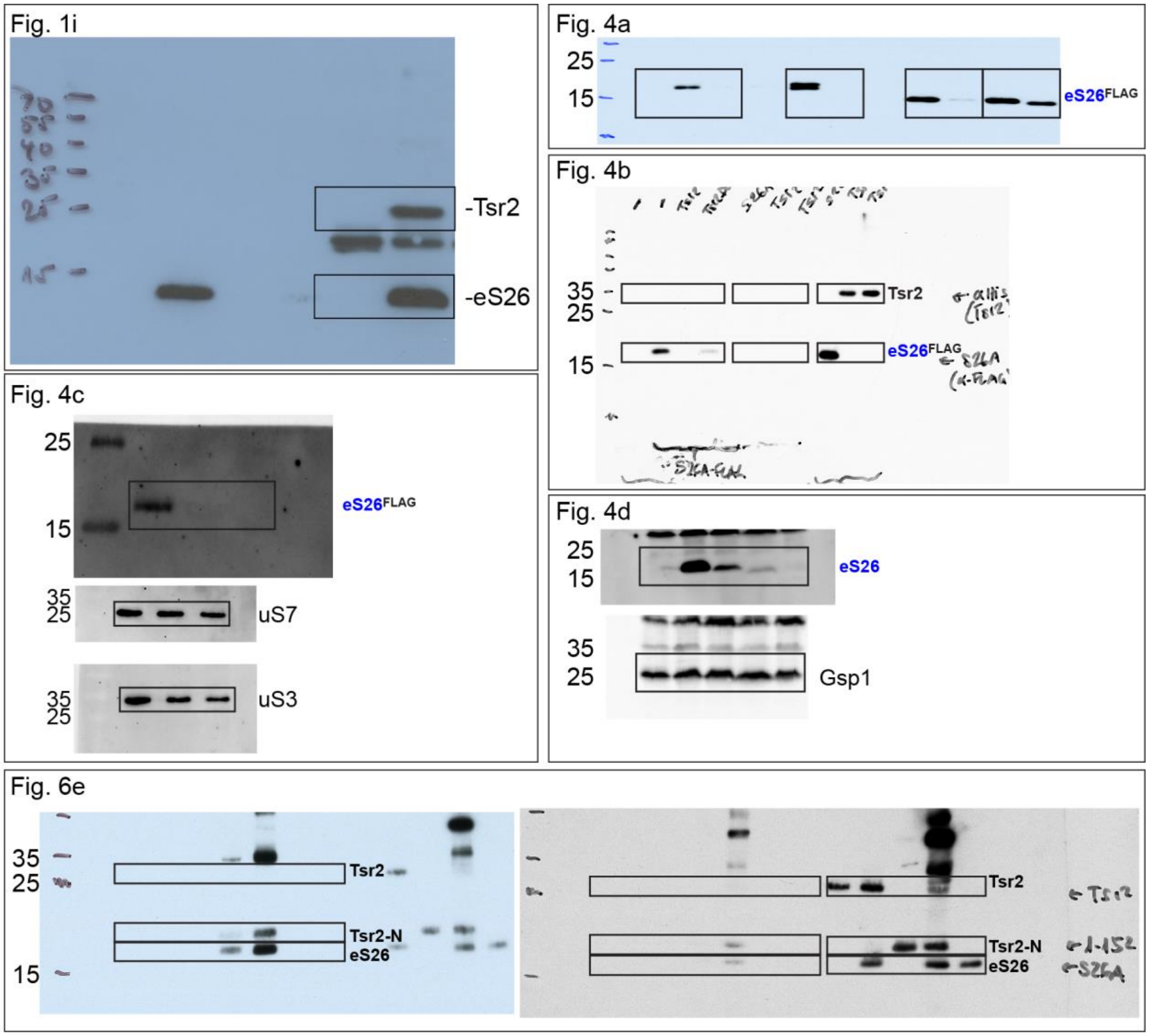

Supplementary Figure 8

Uncropped Western blots from main figures

Supplementary Table 1. XL-MS crosslinks between eS26 and Tsr2

Id	Protein1	Protein2	XLType	Abs Pos 1	Abs Pos 2	Id-Score	Cross-linker	Protease	Instrument
TNLMFSDEKQQAR-VSPADAAKK-a9-b8	sp|Q06672|TSR2_YEAST	sp|P39938|RS26A_YEAST	**inter-protein xl**	27	116	**35.04**	DSS	trypsin	OT Elite
LYLEWQEKQR-GHVKPVR-a8-b4	sp|Q06672|TSR2_YEAST	sp|P39938|RS26A_YEAST	**inter-protein xl**	138	19	**32.56**	DSS	trypsin	OT Elite
LYLEWQEKQR-VSPADAAKK-a8-b8	sp|Q06672|TSR2_YEAST	sp|P39938|RS26A_YEAST	**inter-protein xl**	138	116	**26.94**	DSS	trypsin	OT Elite
FELGVSMVIYK-VSPADAAK-a2-b5	sp|Q06672|TSR2_YEAST	sp|P39938|RS26A_YEAST	**inter-protein xl**	33	113	**27.82**	PDH	trypsin	OT Elite
FNRENKVSPADAAK-FELGVSMVIYK-a6-b2	sp|P39938|RS26A_YEAST	sp|Q06672|TSR2_YEAST	**inter-protein xl**	108	33	**31.36**	PDHZL	trypsin	OT Elite
ENKVSPADAAK-FELGVSMVIYK-a3-b2	sp|P39938|RS26A_YEAST	sp|Q06672|TSR2_YEAST	**inter-protein xl**	108	33	**29.63**	PDHZL	trypsin	OT Elite
FELGVSMVIYK-VSPADAAKK-a2-b8	sp|Q06672|TSR2_YEAST	sp|P39938|RS26A_YEAST	**inter-protein xl**	33	116	**26.73**	PDHZL	trypsin	OT Elite
WDALDVAVENSWGGPDSAEK-ENKVSPADAAK-a16-b3	sp|Q06672|TSR2_YEAST	sp|P39938|RS26A_YEAST	**inter-protein xl**	58	108	**23.55**	PDHZL	trypsin	OT Elite
WDALDVAVENSWGGPDSAEK-ENKVSPADAAK-a19-b3	sp|Q06672|TSR2_YEAST	sp|P39938|RS26A_YEAST	**inter-protein xl**	61	108	**22.21**	PDHZL	trypsin	OT Elite
NIVEAAAVR-LYLEWQEK-a4-b7	sp|P39938|RS26A_YEAST	sp|Q06672|TSR2_YEAST	**inter-protein xl**	46	137	**27**	ADH	trypsin	OT XL
NIVEAAAVR-LYLEWQEK-a4-b4	sp|P39938|RS26A_YEAST	sp|Q06672|TSR2_YEAST	**inter-protein xl**	46	134	**19.14**	ADH	trypsin	OT XL
VEKLYLEWQEK-GHVKPVR-a3-b4	sp|Q06672|TSR2_YEAST	sp|P39938|RS26A_YEAST	**inter-protein xl**	130	19	**22.77**	DSS	trypsin	OT XL
TNLMFSDEKQQAR-VSPADAAKK-a9-b8	sp|Q06672|TSR2_YEAST	sp|P39938|RS26A_YEAST	**inter-protein xl**	27	116	**21.18**	DSS	trypsin	OT XL
CVNCSKSIPK-DKAIK-a6-b2	sp|P39938|RS26A_YEAST	sp|P39938|RS26A_YEAST	intra-protein xl	28	34	**33.8**	DSS	trypsin	OT Elite
TYNKLHYCVSCAIHAR-GHVKPVR-a4-b4	sp|P39938|RS26A_YEAST	sp|P39938|RS26A_YEAST	intra-protein xl	70	19	**32.44**	DSS	trypsin	OT Elite
TYNKLHYCVSCAIHAR-GRGHVKPVR-a4-b6	sp|P39938|RS26A_YEAST	sp|P39938|RS26A_YEAST	intra-protein xl	70	19	**26.09**	DSS	trypsin	OT Elite
TAFVQAEQGKTNLMFSDE-KQQARFE-a10-b1	sp|Q06672|TSR2_YEAST	sp|Q06672|TSR2_YEAST	intra-protein xl	18	27	**29.59**	DSS	Glu-C	OT Elite
MDEVVPDLVSSKPEPIVD-EDGFELVQPKGRRKH-a12-b14	sp|Q06672|TSR2_YEAST	sp|Q06672|TSR2_YEAST	intra-protein xl	184	204	**24.99**	DSS	Glu-C	OT Elite
KQQARFE-KLYLE-a1-b1	sp|Q06672|TSR2_YEAST	sp|Q06672|TSR2_YEAST	intra-protein xl	27	130	**22.53**	DSS	Glu-C	OT Elite

QGKTNLMFSDE-KQQARFE-a10-b1	sp|Q06672|TSR2_YEAST	sp|Q06672|TSR2_YEAST	intra-protein xl	25	27	**25.09**	PDHZL	Glu-C	OT Elite
NIVEAAAVR-VSPADAAK-a4-b5	sp|P39938|RS26A_YEAST	sp|P39938|RS26A_YEAST	intra-protein xl	46	113	**22.91**	ADH	trypsin	OT XL
ALDYKDDDDK-VSPADAAKK-a5-b8	sp|P39938|RS26A_YEAST	sp|P39938|RS26A_YEAST	intra-protein xl	122	116	**28.72**	DSS	trypsin	OT XL
TYNKLHYCVSCAIHAR-GHVKPVR-a4-b4	sp|P39938|RS26A_YEAST	sp|P39938|RS26A_YEAST	intra-protein xl	70	19	**23.14**	DSS	trypsin	OT XL
FNRENKVSPADAAK-KALDYKDDDDK-a6-b1	sp|P39938|RS26A_YEAST	sp|P39938|RS26A_YEAST	intra-protein xl	108	117	**21.82**	DSS	trypsin	OT XL
CVNCSKSIPK-GRGHVKPVR-a6-b6	sp|P39938|RS26A_YEAST	sp|P39938|RS26A_YEAST	intra-protein xl	28	19	**21.42**	DSS	trypsin	OT XL
ENKVSPADAAK-KALDYKDDDDK-a3-b1	sp|P39938|RS26A_YEAST	sp|P39938|RS26A_YEAST	intra-protein xl	108	117	**18.04**	DSS	trypsin	OT XL
TYNKLHYCVSCAIHAR-SIPKDK-a4-b4	sp|P39938|RS26A_YEAST	sp|P39938|RS26A_YEAST	intra-protein xl	70	32	**22.18**	DSS	trypsin	OT XL
ENKVSPADAAK-KALDYK-a3-b1	sp|P39938|RS26A_YEAST	sp|P39938|RS26A_YEAST	intra-protein xl	108	117	**16.56**	DSS	trypsin	OT XL
FNRENKVSPADAAK-KALDYK-a6-b1	sp|P39938|RS26A_YEAST	sp|P39938|RS26A_YEAST	intra-protein xl	108	117	**17.39**	DSS	trypsin	OT XL
ENKVSPADAAK-KALDYKDDDDK-a3-b6	sp|P39938|RS26A_YEAST	sp|P39938|RS26A_YEAST	intra-protein xl	108	122	**18.31**	DSS	trypsin	OT XL
CVNCSKSIPK-GHVKPVR-a6-b4	sp|P39938|RS26A_YEAST	sp|P39938|RS26A_YEAST	intra-protein xl	28	19	**17.64**	DSS	trypsin	OT XL
TYNKLHYCVSCAIHAR-GRGHVKPVR-a4-b6	sp|P39938|RS26A_YEAST	sp|P39938|RS26A_YEAST	intra-protein xl	70	19	**18.61**	DSS	trypsin	OT XL

**Description of the column headers**	
Id	Assigned peptides and cross-linking sites within the *peptide* sequences. The longer peptide is designated as (a)lpha, the shorter as (b)eta.
Protein1	SwissProt/UniProt accession number and identifier of the protein 1 (containing peptide desginated as alpha).
Protein2	SwissProt/UniProt accession number and identifier of the protein 2 (containing peptide desginated as beta).
XLType	Intra- or inter-protein, sometimes also ambiguous.
AbsPos1	Position in the *protein* sequence of protein 1.
AbsPos2	Position in the *protein* sequence of protein 2.
Id-score	Identification score as assigned by xQuest. The higher, the better.

Supplementary Table 2. Input for the structure calculation and characterization of the 20 energy-minimized NMR structures of TSR2-N

Parameter	Value[a]
NOE upper distance limits	3818
Intra-residual	918
Short-range	1058
Medium-range	1071
Long-range	771
Dihedral angle constraints (ϕ, ψ, χ^1 and χ^2)	521
Residual target function (Å^2)	2.23 ± 0.08
Residual distance constraint violations	
Number ≥ 0.2 Å	3 ± 1.3
Maximum (Å)	0.32 ± 0.10
Residual dihedral angle constraint violations	
Number ≥ 2.5°	0.0 ± 0.0
Maximum (°)	0.69 ± 0.31
Mean AMBER energy (kcal/mol)	-4265 ± 17
RMSD from ideal geometry	
Bond lengths (Å)	0.0037 ± 0.0000
Bond angles (°)	1.436 ± 0.008
RMSD to the mean coordinates (Å)[b]	
Backbone heavy atoms	0.50 ± 0.12
All heavy atoms	0.89 ± 0.13
Ramachandran plot statistics (%)[c]	
Most favored regions	85.8 ± 0.8
Additionally allowed regions	13.9 ± 0.8
Generously allowed regions	0.3 ± 0.4
Disallowed regions	0.0 ± 0.0

[a] Except for the top six entries, the data characterize 20 CYANA conformers after energy-minimization with AMBER that are used to represent the NMR structure; the mean values and standard deviations are given.
[b] Backbone heavy atoms include N, C$^\alpha$ and C'. The RMSD values are calculated for residues 10–140.
[c] As determined by PROCHECK.

Supplementary Table 3. Input for the structure calculation and characterization of the 20 energy-minimized NMR structures of TSR2-N in complex with ESS2

Parameter	Value[a]
NOE upper distance limits	4418
Tsr2-N	3569
Intra-residual	831
Short-range	923
Medium-range	937
Long-range	878
ESS2	518
Intra-residual	175
Short-range	183
Medium-range	159
Long-range	1
Complex Tsr2-N:ESS2	
intermolecular	331
Dihedral angle constraints (ϕ, ψ, χ^1 and χ^2)	601
Tsr2-N	521
ESS2	80
Residual target function (Å^2)	3.06 ± 0.22
Residual distance constraint violations	
Number ≥ 0.2 Å	12.0 ± 2.5
Maximum (Å)	0.42 ± 0.08
Mean AMBER energy (kcal/mol)	-5344.3 ± 20.1
RMSD from ideal geometry	
Bond lengths (Å)	0.0037 ± 0.0001
Bond angles (°)	1.482 ± 0.016
RMSD to the mean coordinates (Å)[b]	
Tsr2-N	
Backbone heavy atoms	0.48 ± 0.10
All heavy atoms	0.98 ± 0.13
ESS2	
Backbone heavy atoms	0.18 ± 0.06
All heavy atoms	0.80 ± 0.23
Complex Tsr2-N:ESS2	
Backbone heavy atoms	0.46 ± 0.09
All heavy atoms	0.97 ± 0.12
Ramachandran plot statistics (%)[c]	
Most favored regions	90.9 ± 1.5
Additionally allowed regions	9.1 ± 1.5
Generously allowed regions	0.0 ± 0.0
Disallowed regions	0.0 ± 0.0

[a] Except for the top six entries, the data characterize 20 CYANA conformers after energy-minimization with AMBER that are used to represent the NMR structure; the mean values and standard deviations are given.

[b] Backbone heavy atoms include N, C^{α} and C'. The RMSD values are calculated for residues 10–140 of Tsr2-N and residues 100–119 of ESS2.
[c] As determined by PROCHECK.

Supplementary Table 4. Yeast strains used in this study

**Strain name**	**Genotype**	**Origin**
BY4741	*MATa ura3 his3 leu2 met15 TRP1*	Euroscarf
NMY32	*MATa trp1 leu2 (lexAop)8-ADE2 LYS2::(lexAop)4-HIS3 URA3::(lexAop)8-lacZ GAL4*	Dualsystems Biotech AG
Enp1-TAP	*MATa ura3 leu2 TRP1 ENP1-TAP::HIS3MX*	(Schütz et al., 2014)[4]
P_{GAL1}-*TSR2*	*MATa ura3 his3 leu2 met15 TRP1 Gal1-TSR2::natNT2*	(Schütz et al., 2014)
P_{GAL1}-*RPS26A rps26b*Δ	*MATa ura3 leu2 met15 TRP1 Gal1-RPS26A::natNT2 RPS26B::HIS3MX*	(Schütz et al., 2014)
Enp1-TAP P_{GAL1}-*RPS26A rps26b*Δ	*MATa ura3 leu2 met15 TRP1 Gal1-RPS26A::natNT2 RPS26B::HIS3MX ENP1-TAP::kanMX6*	this study

Supplementary Table 5. Plasmids used in this study

pRS425-*TSR2*	*TSR2 2µ LEU2 AMP*	(Schütz et al., 2014)
pRS425-*TSR2*$^{1\text{-}152}$	*TSR2*$^{1\text{-}152}$ *2µ LEU2 AMP*	this study
pRS425-*TSR2D64AW65AI66A*	*TSR2 D64AW65AI66A 2µ LEU2 AMP*	this study
pRS425-*hTSR2*	*humanTSR2 2µ LEU2 AMP*	this study
pRS425-*hTSR2E64G*	*humanTSR2E64G 2µ LEU2 AMP*	this study
pRS425-*RPS26A*	*RPS26A 2µ LEU2 AMP*	(Schütz et al., 2014)
pRS425-*RPS26AΔeSS1*	*RPS26AΔeSS1 2µ LEU2 AMP*	this study
pRS425-*RPS26AΔeSS2* (pRS425-*RPS26A*$^{1\text{-}99}$)	*RPS26AΔeSS2 2µ LEU2 AMP*	this study
pRS425-*RPS26AΔeSS1ΔeSS2*	*RPS26A ΔeSS1ΔeSS2 2µ LEU2 AMP*	this study
pRS425-*RPS26AΔeSS1*^{FLAG}	*RPS26AΔeSS1 2µ LEU2 AMP*	this study
pRS425-*RPS26AΔeSS2*^{FLAG} (pRS425-*RPS26A*$^{1\text{-}99\text{-}FLAG}$)	*RPS26AΔeSS2 2µ LEU2 AMP*	this study
pRS425-*RPS26AΔeSS1ΔeSS2*^{FLAG}	*RPS26A ΔeSS1ΔeSS2 2µ LEU2 AMP*	this study
pRS425-*RPS26AΔ99-104*	*RPS26AΔ99-104 2µ LEU2 AMP*	this study
pRS425-*FAP7-RPS14A*	*FAP7 RPS1A 2µ LEU2 AMP*	(Peña et al., 2016)[5]
pLexA-dir-*RPS26A*	*LexA-RPS26A 2µ TRP1 AMP*	(Schütz et al., 2014)
pACT-*TSR2*	*GAL4 AD-TSR2 2µ LEU2 KAN*	(Schütz et al., 2014)
pACT-*TSR2*$^{1\text{-}152}$	*GAL4 AD-TSR2*$^{1\text{-}152}$ *2µ LEU2 KAN*	this study
pACT-*TSR2*$^{153\text{-}205}$	*GAL4 AD-TSR2*$^{153\text{-}205}$ *2µ LEU2 KAN*	this study
pACT-*LargeT*	*GAL4 AD-SV40 largeT antigen 2µ LEU2 KAN*	Dual Systems
pETduet1-*RPS26A*	*RPS26A AMP*	(Schütz et al., 2014)
pETduet1-*RPS26A*^{FLAG}	*RPS26A*^{FLAG} *AMP*	(Schütz et al., 2014)

pETduet1-*RPS26AΔeSS1*	*RPS26AΔeSS1 AMP*	this study
pETduet1-*RPS26AΔeSS2*<br>*RPS26A*$^{1-99}$	*RPS26AΔeSS2 AMP*	this study
pETduet1-*RPS26AΔeSS1ΔeSS2*	*RPS26A ΔeSS1ΔeSS2 AMP*	this study
pETduet1-*RPS26AΔeSS1*^{FLAG}	*RPS26AΔeSS1 AMP*	this study
pETduet1-*RPS26AΔeSS2*^{FLAG}<br>pRS425-*RPS26A*$^{1-99}$	*RPS26AΔeSS2 AMP*	this study
pETduet1-*RPS26AΔeSS1ΔeSS2*^{FLAG}	*RPS26A ΔeSS1ΔeSS2 AMP*	this study
pETduet1-*RPS26AΔ99-104*	*RPS26AΔ99-104 AMP*	this study
pETduet1-*RPS26AΔ104-109*	*RPS26AΔ104-109 AMP*	this study
pETduet1-*RPS26AΔ109-114*	*RPS26AΔ109-114 AMP*	this study
pETduet1-*RPS26AΔ114-119*	*RPS26AΔ114-119 AMP*	this study
pETduet1-*RPS26AΔ99-109*	*RPS26AΔ99-109 AMP*	this study
pETduet1-*RPS26AΔ104-114*	*RPS26AΔ104-114 AMP*	this study
pETduet1-*RPS26AΔ109-119*	*RPS26AΔ109-119 AMP*	this study
pETduet1-*RPS26A*$^{1-104}$	*RPS26A*$^{1-104}$ *AMP*	this study
pETduet1-*RPS26A*$^{1-109}$	*RPS26A*$^{1-109}$ *AMP*	this study
pETduet1-*RPS26A*$^{1-114}$	*RPS26A*$^{1-114}$ *AMP*	this study
pETduet1-*hRPS26A*^{FLAG}	*humanRPS26A*^{FLAG} *AMP*	this study
pETduet1-*HIS*$_6$-*TSR2*	*HIS*$_6$-*TSR2 AMP*	(Schütz et al., 2014)
pETduet1-*HIS*$_6$-*TSR2*$^{1-152}$	*HIS*$_6$-*TSR2*$^{1-152}$ *AMP*	this study
pETduet1-*HIS*$_6$-*TSR2*<br>*D64AW65AI66A*	*HIS*$_6$-*TSR2D64AW65AI66A AMP*	this study
pETduet1-*HIS*$_6$-*TSR2-RPS26A*	*HIS*$_6$-*TSR2 RPS26A AMP*	(Schütz et al., 2014)
pETduet1-*HIS*$_6$-*TSR2-RPS26A*^{FLAG}	*HIS*$_6$-*TSR2 RPS26A*^{FLAG} *AMP*	(Schütz et al., 2014)
pEM1-*TSR2*	*HIS*$_6$-*GB1-TSR2 AMP*	this study
pEM1-*TSR2*$^{1-152}$	*HIS*$_6$-*GB1-TSR2*$^{1-152}$ *AMP*	this study
pEM1-*TSR2*$^{153-205}$	*HIS*$_6$-*GB1-TSR2*$^{153-205}$ *AMP*	this study
pEM1-*TSR2-RPS26A*	*HIS*$_6$-*GB1-TSR2 RPS26A AMP*	this study
pEM1-*TSR2*$^{1-152}$-*RPS26A*	*HIS*$_6$-*GB1-TSR2*$^{1-152}$ *RPS26A AMP*	this study
pEM1-*RPS26A*$^{99-119}$	*HIS*$_6$-*GB1-RPS26A*$^{99-119}$ *RPS26A AMP*	this study
pGEX-6P-1-*RPS26A*	*GST-RPS26A AMP*	this study
pGEX-6P-1-*TSR2*	*GST-TSR2 AMP*	(Schütz et al., 2014)
pGEX-6P-1-*TSR2*$^{1-152}$	*GST-TSR2*$^{1-152}$ *AMP*	this study
pGEX-6P-1-*TSR2*$^{153-205}$	*GST-TSR2*$^{153-205}$ *AMP*	this study
pGEX-6P-1-*hTSR2*	*humanTSR2 2μ LEU2 AMP*	this study
pGEX-6P-1-*hTSR2E64G*	*humanTSR2E64G 2μ LEU2 AMP*	this study
pGEX-4TEV-*KAP123*	*GST-KAP123 AMP*	(Schütz et al., 2014)

Supplementary References

1. Wishart, D.S. & Sykes, B.D. The 13C chemical-shift index: a simple method for the identification of protein secondary structure using 13C chemical-shift data. *J Biomol NMR* **4**, 171-80 (1994).
2. Ben-Shem, A., Garreau de Loubresse, N., Melnikov, S., Jenner, L., Yusupova, G. & Yusupov, M. The structure of the eukaryotic ribosome at 3.0 A resolution. *Science* **334**, 1524-9 (2011). 10.1126/science.1212642
3. Sievers, F. & Higgins, D.G. Clustal Omega, accurate alignment of very large numbers of sequences. *Methods Mol Biol* **1079**, 105-16 (2014). 10.1007/978-1-62703-646-7_6
4. Schütz, S., Fischer, U., Altvater, M., Nerurkar, P., Pena, C., Gerber, M., Chang, Y., Caesar, S., Schubert, O.T., Schlenstedt, G. & Panse, V.G. A RanGTP-independent mechanism allows ribosomal protein nuclear import for ribosome assembly. *Elife* **3**, e03473 (2014). 10.7554/eLife.03473
5. Pena, C., Schutz, S., Fischer, U., Chang, Y. & Panse, V.G. Prefabrication of a ribosomal protein subcomplex essential for eukaryotic ribosome formation. *Elife* **5**(2016). 10.7554/eLife.21755
